# Metabolomics signatures of depression: the role of symptom profiles

**DOI:** 10.1038/s41398-023-02484-5

**Published:** 2023-06-10

**Authors:** Hilde de Kluiver, Rick Jansen, Brenda W. J. H. Penninx, Erik J. Giltay, Robert A. Schoevers, Yuri Milaneschi

**Affiliations:** 1 Department of Psychiatry, Amsterdam UMC, Vrije Universiteit Amsterdam, Amsterdam, The Netherlands; 2 Amsterdam Public Health, Mental Health program, Amsterdam, The Netherlands; 3 Amsterdam Neuroscience, Mood, Anxiety, Psychosis, Sleep & Stress program, Amsterdam, The Netherlands; 4 Department of Psychiatry, Leiden University Medical Center, Leiden, The Netherlands; 5 University of Groningen, University Medical Center Groningen, Department of Psychiatry and Research School of Behavioural and Cognitive Neurosciences (BCN), Groningen, The Netherlands

**Keywords:** Depression, Diagnostic markers

## Abstract

Depression shows a metabolomic signature overlapping with that of cardiometabolic conditions. Whether this signature is linked to specific depression profiles remains undetermined. Previous research suggested that metabolic alterations cluster more consistently with depressive symptoms of the atypical spectrum related to energy alterations, such as hyperphagia, weight gain, hypersomnia, fatigue and leaden paralysis. We characterized the metabolomic signature of an “atypical/energy-related” symptom (AES) profile and evaluated its specificity and consistency. Fifty-one metabolites measured using the Nightingale platform in 2876 participants from the Netherlands Study of Depression and Anxiety were analyzed. An ‘AES profile’ score was based on five items of the Inventory of Depressive Symptomatology (IDS) questionnaire. The AES profile was significantly associated with 31 metabolites including higher glycoprotein acetyls (β = 0.13, *p* = 1.35*10^-12^), isoleucine (β = 0.13, *p* = 1.45*10^-10^), very-low-density lipoproteins cholesterol (β = 0.11, *p* = 6.19*10^-9^) and saturated fatty acid levels (β = 0.09, *p* = 3.68*10^-10^), and lower high-density lipoproteins cholesterol (β = −0.07, *p* = 1.14*10^-4^). The metabolites were not significantly associated with a summary score of all other IDS items not included in the AES profile. Twenty-five AES-metabolites associations were internally replicated using data from the same subjects (*N* = 2015) collected at 6-year follow-up. We identified a specific metabolomic signature—commonly linked to cardiometabolic disorders—associated with a depression profile characterized by atypical, energy-related symptoms. The specific clustering of a metabolomic signature with a clinical profile identifies a more homogenous subgroup of depressed patients at higher cardiometabolic risk, and may represent a valuable target for interventions aiming at reducing depression’s detrimental impact on health.

## Introduction

Depression often co-occurs with cardiometabolic conditions (obesity, type 2 diabetes, cardiovascular diseases) [1–3] and shared underlying biological pathways may partially explain this connection. A large-scale metabolomics meta-analysis on >15,000 subjects [4] showed that depression is associated with a distinctive “immuno-metabolic” signature (e.g., higher glycoprotein acetyls, triglycerides and very-low-density lipoproteins (VLDL), and lower high-density lipoproteins (HDL)) that overlaps with that of cardiometabolic conditions [5–7], representing therefore a potential connecting substrate and a promising target for intervention. Whether this signature is similarly associated with all clinical manifestations of depression or more strongly linked to specific clinical profiles remains undetermined.

Recent large-scale meta-analyses and biobank studies [8, 9] showed that higher blood levels of inflammatory markers such as C-reactive protein (CRP) and interleukin-6 (IL-6) were specifically associated with depressed mood, anhedonia and somatic symptoms such as changes in appetite, sleep alterations and fatigue. Cohort and case-control studies [10–13] with more refined assessment tools of somatic symptoms indicated that those pertaining to the “atypical” spectrum—in particular the reversed neurovegetative symptoms of hyperphagia, hypersomnia and weight gain together with leaden paralysis—were more strongly linked to markers of inflammatory and metabolic dysregulations including CRP, IL-6, triglycerides, leptin, insulin and different measures of body fat and adiposity. Large-scale genomic studies [14, 15] identified shared genetic liabilities between symptoms of hyperphagia, hypersomnia and weight gain during a depressive episode and traits such as elevated CRP, leptin, and body mass index (BMI), partially explaining their phenotypic connection. Previous analyses [16, 17] in the Netherlands Study of Depression and Anxiety cohort combined the “atypical” symptoms of hyperphagia, hypersomnia, weight gain, fatigue and leaden paralyses measured with a self-report questionnaire in a dimensional profile labelled “atypical, energy-related” symptoms (AES); the studies showed that only the AES profile was specifically associated with markers of inflammation (CRP, IL-6, neurotoxic tryptophan catabolites of the indoleamine pathways) and metabolic syndrome (triglycerides, HDL cholesterol, glucose, waist circumference, blood pressure), while no such associations were found for profiles capturing melancholic or anxious-distress symptoms. The specific association of CRP and IL-6 with AES, but not with melancholic or anxious-distress symptom profiles, was replicated in a study [18] of 158 depressed patients. The covariance of this type of atypical-like depressive symptoms with inflammatory and metabolic dysregulations has been postulated to identify a dimension labelled “immuno-metabolic depression” [19], which map the degree of expression of behavioral and biological processes overlapping with those in cardiometabolic phenotypes.

While previous analyses explored the biological signature of this set of “atypical, energy-related symptoms” with limited selective biomarkers, the present study aims to (1) identify in a large sample the biological signature of the AES depression profile using a large metabolomics panel, and (2) to extensively evaluate its specificity in relation to different symptom profiles and explanatory factors (e.g. severity, diagnostic status, antidepressant use), and (3) its consistency by performing an internal replication with data from the same subjects collected 6 years later.

## Materials and methods

### Sample

Subjects were from the Netherlands Study for Depression and Anxiety (NESDA), an ongoing longitudinal cohort study into the long-term course and consequences of depressive and anxiety disorders. A description of the study rationale, design, and methods is given elsewhere [20]. In 2004–2007, participants aged 18–65 years were recruited from the community (19%), general practice (54%) and secondary mental health care (27%). A total of 2981 participants were included, consisting of persons with current (i.e., within 6 months prior to interview) or past DSM-IV diagnoses of depressive (major depressive disorder, dysthymia) and/or anxiety (social phobia, generalized anxiety disorder, panic disorder, agoraphobia) disorder ascertained with the Composite Interview Diagnostic Instrument (CIDI) version 2.1 [21], and healthy controls (i.e., no lifetime psychiatric diagnosis). Participants were not included when they could not speak Dutch fluently or had another primary psychiatric diagnosis of e.g. bipolar, psychotic, obsessive compulsive or severe addictive disorder. The Ethical Committee of participating universities approved the NESDA project and all participants provided written informed consent. Participants were evaluated at baseline and followed-up during biannual assessments including an extensive interview, medical assessment, blood collection and self-reported questionnaires. For the main analyses, we selected 2876 participants from the baseline assessment with data on depressive symptoms and metabolomics. Furthermore, we performed internal replication analyses with data measured at 6-year follow-up (2010-2013) in 2015 subjects. At the 6-year follow-up, similar procedures were used to collect plasma samples, and measure metabolomics, depression symptoms and diagnoses and covariates. Thus, measurements described in the next sections apply to both baseline and 6-year follow-up assessments.

### Metabolomic markers measurement

Fasting plasma samples were obtained in the morning and stored at -80 °C. Metabolomic profiles were measured using a proton Nuclear Magnetic Resonance (NMR) platform [22] by Nightingale Health Ltd., Helsinki, Finland. Baseline samples were measured in two batches at different times (further referred to as batch 1 and 2, respectively) and a previous study [23] flagged eleven metabolites with different levels across batches. Thus, all analyses of baseline data were adjusted for assessment batch. At 6 years, all samples were analyzed in one batch. The metabolomic platform quantified 51 metabolites including 8 amino acids, 2 apolipoproteins, 9 cholesterol measures, 8 fatty acids, 2 fluid balance related measures, 9 glycerides and phospholipids, 3 glycolysis-related metabolites, 1 inflammation-related metabolite, 3 ketone bodies, 3 lipoprotein particle sizes, and 3 total fatty acids and saturation measures. The NMR platform includes additional sub measures and ratios of these lipoprotein (i.e., 98 lipid composition and particle concentration measures of lipoprotein subclasses and 81 lipid and fatty acids ratios), which were not included in the current analyses because of redundancy of information. The raw metabolite variables were prepared for analyses according to a standardized protocol suggested by the manufacturer and as used before [4]. A value of 1 was added to each value, after which we applied a natural log transformation. Metabolite values 5 SD above or below the mean were truncated at the 5 SD levels.

### Atypical, energy-related symptom (AES) profile

Depressive symptoms endorsed in the week before the interview and their severity were measured with the Inventory of Depressive Symptomatology (IDS) [24], rating the core symptoms of a major depressive episodes, melancholic and atypical features, and commonly associated (somatic) complaints. An atypical, energy-related depressive symptom (AES) profile was created in the full sample as described in previous studies [16, 17] by summing the scores on five items: leaden paralysis, energy loss, hypersomnia, hyperphagia, and increased weight. The AES profile ranges from 0 (not severe) to 15 (severe). The mean inter-item Spearman correlation for the AES profile was 0.25, indicating a satisfactory level [25] of homogeneity. For comparison purpose we derived a summary score including all other IDS items not included in the AES profile (“other-IDS symptoms severity”, see Supplementary Methods for details).

### Covariates

Sex, age, education level (years), and current smoking status were assessed as part of the interview. The number of self-reported chronic diseases for which persons received treatment (including cardiovascular disorders, diabetes, lung disease, osteoarthritis, cancer, ulcer, intestinal or liver diseases, epilepsy, and thyroid gland disease) was calculated as a global marker of poor physical health. BMI (kg/m^2^) was calculated from weight and height measures. The use of medication was based on container inspection of drugs used in the past month and classified according to the Anatomical Therapeutic Chemical (ATC) classification system. Selected medications were considered when taken on a regular basis (at least 50% of the time) and included: C10 lipid-modifying agents, N06AB selective serotonin reuptake inhibitors (SSRI), N06AA tricyclic antidepressants (TCA) and N06AX other antidepressants. Overnight fasting status (yes/no) at time of blood withdrawal was documented. For baseline data, metabolomics assessment batch (1 or 2) was also documented.

### Statistical analyses

Variables were reported as percentages or means ± standard deviation as appropriate. In order to identify its metabolomic signature (aim 1), the AES profile was separately regressed on each metabolite adjusting for age, sex, education level, smoking status, fasting status, number of somatic diseases, assessment batch (for baseline data), and lipid lowering drug use. A separate model tested the impact of additionally adjusting for BMI, which has been shown [7] to be associated with multiple markers of the platform. Multiple testing correction taking into account intercorrelations between markers and minimizing the chance of detecting false positives was done based on permutations combined with False Discovery Rate (FDR) computation. We permuted the order of the 51 metabolomic markers, while keeping the order of the AES score and all covariates included in the model in place. We did this 100,000 times and reran the statistical models on the permuted data. We computed the False Discovery Rate (FDR) for each marker by dividing the average number of *p-*values (per permutation) obtained from the analyses run on the permuted data lower than or equal to the real *p*-value of this marker by the total number of real *p*-values lower than or equal to the real *p-*value of this marker. An FDR < 5% was considered as statistically significant. We further examined the coherency of the of symptoms included in the AES profile in additional analyses estimating their individual association with metabolites.

To examine the specificity of the associations detected (aim 2), we plotted the effect size of the AES profile-metabolite associations against the effect sizes obtained regressing the other-IDS symptoms severity on metabolites. To further establish the specificity of the associations between the AES profile and metabolites, we also examined whether these associations were more likely to be significant than associations between metabolomic markers and any other possible score made out of five other IDS items that did not belong to AES (see Supplementary Methods for details). Furthermore, we examined the impact of antidepressant medications on established associations. We firstly identified metabolites associated with antidepressants use in the >1000 drug-metabolite associations listed in the atlas built on >18,000 subjects by BBMRI-NL [26]. Then, we re-estimated the association between those metabolites and AES profile in non-antidepressant users. Finally, in order to further rule out the possibility that factors such as severity or diagnostic status may have impacted on the associations detected we performed specific analyses focusing on a selected homogenous subset of subjects with a depression diagnosis in the last month and moderate to severe symptoms based on IDS ≥ 26 [27]. These cases were stratified according to the median AES profile score in a “low” (AES score < 6, *N* = 310) and a “high” (AES score ≥ 6, *N* = 355) group. For this analyses we further selected 563 healthy controls (80% of those initially available) with IDS < 14 (*n* = 563), indicating complete absence of current depressive symptoms [27]. Mean levels of metabolites across these depression groups and controls were compared.

In order to evaluate the consistency (aim 3) of the significant associations between AES symptoms and metabolites we tested their replication in data collected at the 6-year follow-up.

Finally, in exploratory analyses on baseline data we applied Least Absolute Shrinkage and Selection Operator (LASSO) analyses (λ regularization using a 10-fold cross-validation) to select among a wide array of correlated markers, a more parsimonious set of metabolites describing the AES profile.

All statistical analyses were conducted with the use of R software version 3.6.0(R Core Team, 2019).

## Results

Table 1 presents baseline characteristics of the analytical sample. The mean age was 42.0 ± 13.0 years and 66.6% of the participants were female. More than half of the sample (57.1%) had a current depressive and/or anxiety disorder. The mean IDS score was 21.5 ± 14.1.

**Table 1:** Baseline characteristics of the study sample.

Baseline characteristics	Analytical sample
*N*	2876
*Sociodemographic variables*	
Age, mean (sd)	42.0 (13.0)
Female, *N* (%)	1915 (66.6)
Years of education, mean (sd)	12.2 (3.3)
*Lifestyle and somatic health variables*	
Current smoker, *N* (%)	1102 (38.3)
BMI, mean (sd)	25.6 (4.9)
Number of somatic diseases, mean (sd)	0.9 (1.1)
Lipid lowering drug use, *N* (%)	202 (7.0)
*Clinical characteristics*	
Current depression and/or anxiety, *N* %	1641 (57.1)
Remitted depression and/or anxiety, *N* (%)	604 (21.9)
Healthy control, *N* (%)	631 (21.0)
Total IDS, mean (sd)	21.5 (14.1)
Antidepressant use, *N* (%)	709 (24.7)
*Depression severity variables*	
AES profile, mean (sd)	3.3 (2.7)
Other-IDS symptoms severity, mean (sd)	18.2 (12.2)
*Blood sampling variables*	
Overnight fasting at time of blood draw, *N* (%)	2745 (95.4)
Assessed in batch 1, %	1310 (45.5)

*BMI* Body Mass Index, *IDS* Inventory of Depressive Symptomatology, *AES* atypical, energy-related symptoms.

**Fig. 1:**
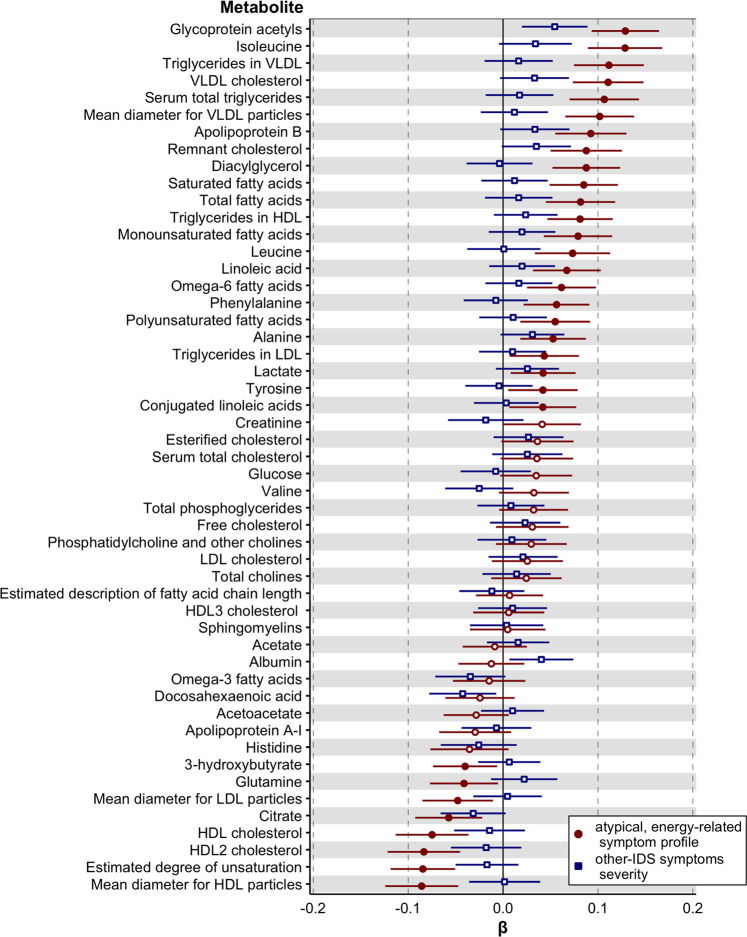
Baseline associations of 51 metabolomic markers with the atypical, energy symptom profile and with the other-IDS symptoms severity. Standardized estimates and 95% confidence intervals from linear regression models. Red: atypical, energy-related symptom profile; blue: other-IDS symptoms severity. Filled indicators indicate significant associations at False Discovery Rate <5%.

**Fig. 2:**
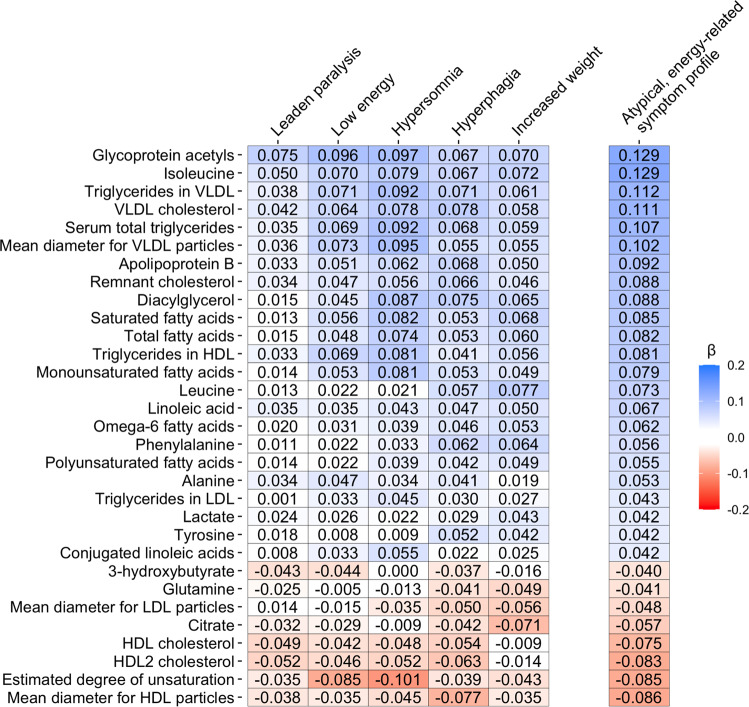
Associations between individual atypical, energy-related symptoms and 31 metabolomic markers. Standardized estimates from linear regression models. Five left columns: individual atypical, energy-related symptoms; right column: atypical, energy-related symptom profile.

We examined the association of AES profile with metabolites (aim 1) adjusting for age, sex, education level, smoking status, fasting status, number of somatic diseases, assessment batch, and lipid lowering drug use. Out of 51 tested markers, 31 were significantly (permutation-based FDR < .05) associated with the AES profile (Fig. 1, full results in Supplementary Table 1). The five top ranking markers were metabolites belonging to the classes of inflammation (glycoprotein acetyls; β = 0.13, se = 0.02, *p* = 1.35*10^-12^), amino acids (isoleucine: β = 0.13, se = 0.02, *p* = 1.45*10^-10^), glycerides and phospholipids (triglycerides in VLDL: β = 0.11, se = 0.02, *p* = 3.26*10^-9^ and serum total triglycerides: β = 0.11, se = 0.02, *p* = 1.20*10^-8^), and cholesterol (VLDL cholesterol: β = 0.11, se = 0.02, *p* = 6.19*10^-9^). After additional adjustment for BMI, the effect sizes of the associations were attenuated, but 12 metabolites were still significantly associated with the AES profile, including the five top ranking markers found in the main model. We further explored the associations between the individual AES symptoms and the 31 metabolites linked to the AES profile (see heatmap in Fig. 2; Supplementary Table 2 for full results). For each metabolite, directions of effect sizes were highly consistent across AES symptoms, indicating that these five symptoms have converging biological associations and AES profile associations are not just driven by a single symptom only.

We subsequently examined the specificity of the associations detected. Figure 1 additionally illustrates the estimates of the associations of metabolites with the other-IDS symptoms severity. The other-IDS symptoms severity was not significantly associated with any of the metabolites (FDR > 0.05, Supplementary Table 3), and estimates were consistently stronger for the AES profile, suggesting a specific link between the latter symptoms and metabolites not spuriously impacted by generic symptom severity. Specificity of the association between the AES profile and metabolites was confirmed by comparing their statistical significance with that of associations between metabolites and any of the other 53130 potential random five IDS-symptom combinations. The associations between the AES profile with 24 out of the 31 previously identified markers (Supplementary Table 4) were statistically more significant (FDR < 0.05) than any random potential symptom combinations.

Furthermore, we examined the potential impact of antidepressant use on the detected associations. Among the 31 markers selected, the BBMRI-NL atlas [26] included associations of: (a) VLDL cholesterol and apolipoprotein B with venlafaxine; (b) estimated degree of unsaturation of fatty acids, HDL and HDL2 cholesterol with SSRIs. In analyses focusing on non-venlafaxine users (*N* = 2 767), VLDL cholesterol (β = 0.10, se = 0.02, *p* = 1.81*10^-7^) and apolipoprotein B (β = 0.08, se = 0.02, *p* = 2.38*10^-5^) were significantly associated with AES profile. In non-SSRI users (*N* = 2,388), HDL cholesterol (β = −0.05, se=0.02, *p* = 0.02), HDL2 cholesterol (β = −0.06, se = 0.02, *p* = 8.01*10^-3^) and estimated degree of fatty acids unsaturation (β = −0.06, se=0.02, *p* = 1.58*10^-3^) were significantly associated with AES profile. Thus, results were very similar to those from the main analyses, implying that the significant associations were not substantially explained by antidepressant use. In additional sensitivity analyses examining the potential impact of comorbid disease, we re-estimated the association of metabolites with AES in 1665 subjects (57.9% of the sample) without comorbid somatic diseases. Results were substantially unchanged (Supplementary Table 5).

**Fig. 3:**
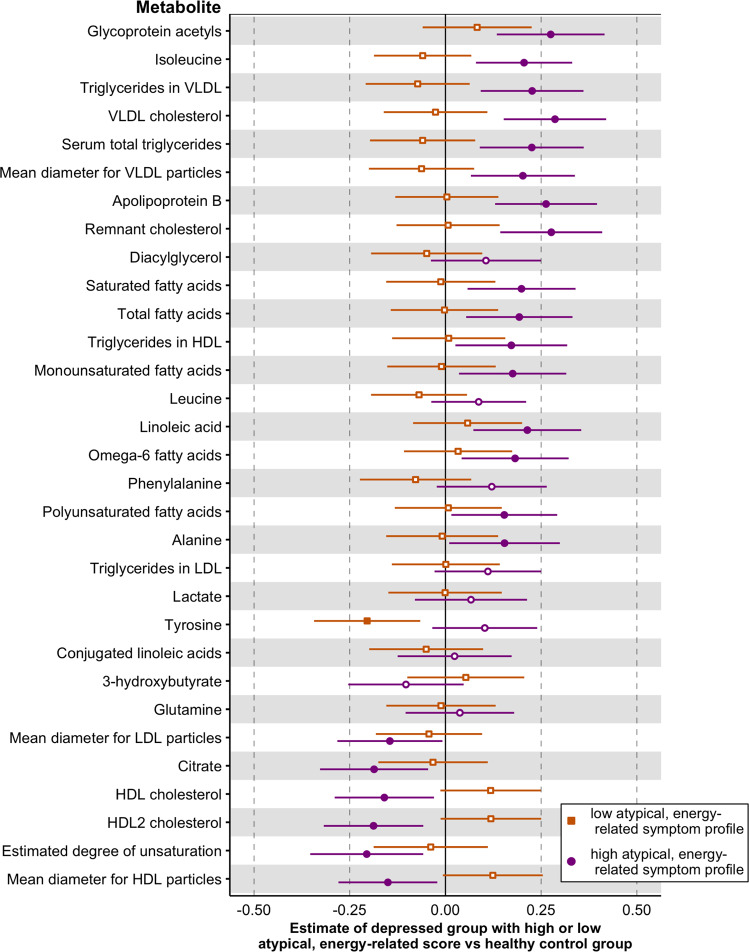
Metabolite levels difference of depressed cases with low and high atypical, energy-related symptom profiles versus healthy controls. Estimated mean differences and 95% confidence intervals of metabolite levels from general linear model. Reference group: healthy controls. Depressed cases stratified in high (purple indicators) and low (orange indicators) atypical, energy-related symptom profile scores. Filled indicators indicate a significant difference (*p* < .05) from healthy controls.

Next, we examined the potential impact on the associations between depressive symptoms and metabolites of the composition of our analytical sample, including subjects at different stages of depression and anxiety disorders. Thus, we focused on an a highly selected and homogenous subset of subjects with current depressive disorders in the last month and moderate to severe symptomatology stratified in a “low” (mean IDS 35.8 ± 7.3) and “high” (mean IDS 42.2 ± 8.9) AES score groups. Levels of 31 metabolites were compared across these subgroups and healthy controls without current depressive symptoms (Fig. 3; full results in Supplementary Table 6). For the majority of markers (22 out of 31), depressed persons with a high AES profile differed significantly from controls, whereas depressed persons with a low AES profile were not significantly different from healthy controls.

**Fig. 4:**
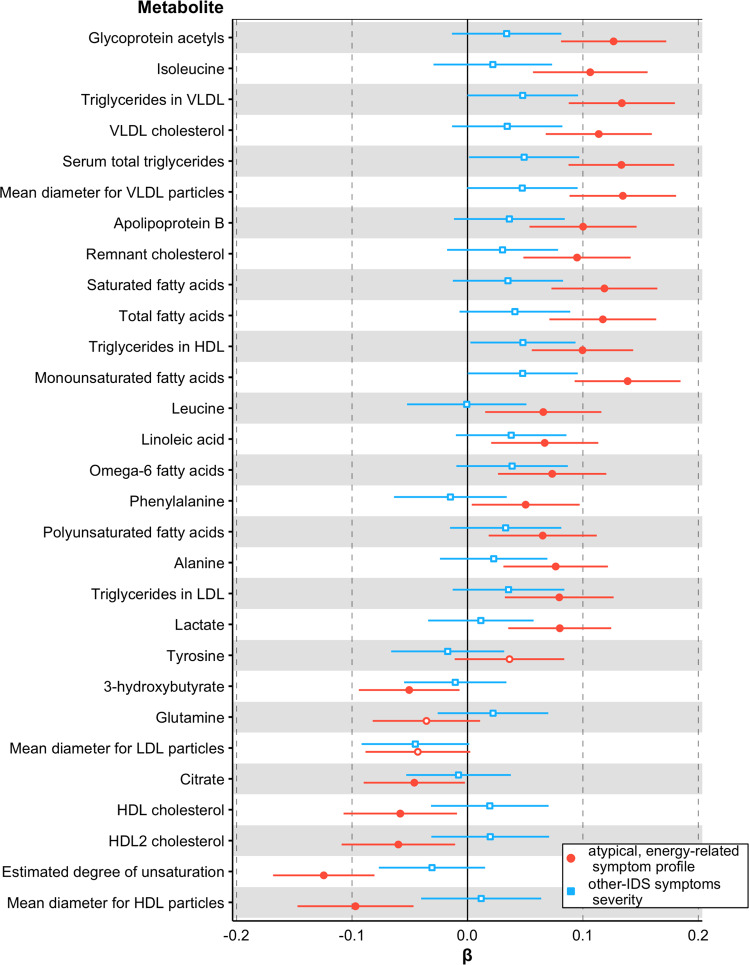
Six-year follow-up associations of 31 metabolomic markers with the atypical, energy symptom related profile and with the other-IDS symptoms severity. Standardized estimates and 95% confidence intervals from linear regression models. Red: atypical, energy-related symptom profile; blue: other-IDS items symptoms severity. Filled indicators indicate significant associations at False Discovery Rate <5%.

We tested the consistency of the associations between AES profile and metabolites by checking their replication in data collected on 2015 participants at 6-year follow-up. As compared to baseline, a relatively smaller proportions of subjects had a current depressive and/or anxiety disorder (545, 27.0%) and the mean IDS score was also lower (15.2 ± 11.9). Among the 31 associated metabolites at baseline, 29 were available at follow-up (Fig. 4, Supplementary Table 7) and 25 were significantly associated with AES profile. Association estimates were highly correlated across assessments (*r* = 0.97; Supplementary Fig. 1). Consistently with the main baseline analyses, none of the metabolites was significantly associated with the profile built with the other-IDS symptoms severity, confirming the specificity of the associations previously identified.

In additional exploratory analyses focusing on baseline data, the 31 significantly associated metabolites (Supplementary Fig. 2 shows high cross-metabolite correlations) in main analyses and covariates were included in a LASSO model predicting AES profile score. Among metabolomic markers, the model (explaining a limited portion of AES variance, *r*^*2*^ = 0.18) retained the following markers: glycoprotein acetyls, citrate, HDL2 cholesterol, isoleucine, 3-hydroxybutyrate and estimated degree of unsaturation of fatty acids.

## Discussion

The present large-scale cohort study examined the metabolomic signature associated with the expression of a set of “atypical, energy-related” depressive symptoms (hyperphagia, hypersomnia, weight gain, fatigue and leaden paralyses) previously linked to a limited number of inflammatory and metabolic biomarkers. In the present study, the AES clinical profile was associated with higher levels of glycoprotein acetyls, isoleucine, several VLDL markers and triglycerides, and with lower concentrations of HDL markers and lower degree of unsaturation of fatty acids. A large proportion of the markers associated with AES have been linked to atherosclerosis and insulin resistance processes [28–30]. This immuno-metabolic unfavorable signature is largely in line with the metabolomic profile associated with depression in a previous large meta-analysis [4]. Intriguingly, our analyses clearly suggested that this metabolomic signature seems to be specifically associated with the atypical, energy-related symptom profile as compared to other combinations of different depressive symptoms. This suggests that the association with metabolites is largely dependent on the specific depressive symptoms present and is not a mere consequence of the overall severity of unspecified symptomatology. Furthermore, this specific association was not substantially impacted by other factors such as diagnostic status or antidepressant use. Moreover, this association pattern was internally replicated using data measured again in the same subjects at the 6-year follow-up of the study. Overall, these findings support the hypothesis that immuno-metabolic dysregulations are not uniformly related to all clinical manifestations of depression, but are more strongly linked to specific clinical profiles that could identify patients at higher cardiometabolic risk [19].

Among the measured metabolites, glycoprotein acetyls and isoleucine had the strongest positive association with AES. Glycoprotein acetyls, indexing concentration and glycosylation of several acute phase proteins, is a novel biomarker providing a stable reading of systemic chronic inflammation linked to cardiometabolic health [31]. Isoleucine is a branched-chain amino acid implicated in the etiology of insulin resistance, diabetes and obesity [32]. In previous large-scale studies applying the same metabolomic platform, glycoprotein acetyls and isoleucine were shown to be associated with depression [4] and among the most predictive markers of all-cause mortality [33]. In recent analyses of UKBiobank data [34], both metabolites were associated with a wide plethora of outcomes, including major cardiovascular events and diabetes. This supports the hypothesis of a role for immuno-metabolic dysregulations indexed by these markers as connecting mechanisms between cardiometabolic diseases and depression, in particular for subjects expressing clinical profiles that are characterized by atypical, energy-related symptoms.

The specific association between metabolite alterations and the AES profile may arise from different, mutually non-exclusive, scenarios. In the first scenario, shared sociodemographic (e.g. lower educational attainment), lifestyle (e.g. smoking, alcohol use, sedentariness, poor diet) and health-related (e.g. presence of chronic somatic diseases, use of medications) factors may explain the metabolites-symptoms association. However, adjustment for these major distal factors had a marginal impact on the associations identified, suggesting a potential connection at a more proximal biological level. This idea is consistent with large-scale genomic analyses [14, 15] showing that depressed patients expressing symptoms of hyperphagia, hypersomnia and weight gain during a depressive episode carried a higher genetic loading for immuno-metabolic traits such as elevated BMI, CRP and leptin (i.e., a peptide hormone involved in energy homeostasis [35]). Based on previous research [7], a potentially more relevant role in determining metabolites levels was expected for BMI, whose inclusion in the statistical models partially reduced the strength of the association between AES symptoms and metabolites. Nevertheless, BMI-adjusted estimates should be carefully interpreted due to the complex shared pathways and genetic covariance between BMI, immuno-metabolic dysregulations and atypical-like symptoms. BMI may indeed represent a confounders influencing both metabolic dysregulations and symptoms expressing altered energy intake processes (e.g. increased weight and appetite), but also a consequence (collider) of metabolic alterations affecting both metabolite concentrations and behavioral symptoms (e.g. leptin signaling disruption driving hyperphagia and energy accumulation, ultimately leading to obesity [35]). This scenario is consistent with a recent genetic study [36] showing that the connection between elevated body fat and AES is dependent on the presence of metabolic dysregulations, representing therefore the underlying connecting mechanisms. Therefore, the position of BMI in such complex causal pathways cannot be easily deconvoluted in observational data. Nevertheless, after additional statistical adjustment for BMI the majority of associations detected in the present study—including those with the top-ranking markers of glycoprotein acetyls, isoleucine, triglycerides, and VLDL cholesterol—remained statistically significant. In the second scenario, metabolic alterations may represent the direct consequences of the depressive symptoms hereby examined. For instance, increased intake of high-fat palatable food and reduced physical energy expenditure may result in increased blood concentration of lipids, saturated fatty acids and inflammatory markers. In the third scenario, metabolic alterations may be part of pathophysiological processes leading to the expression of certain symptoms (e.g. inflammation disrupts hypothalamic leptin signaling leading to hyperphagia [37]). Consistently, recent Mendelian Randomization studies [9, 38–41]—leveraging key properties of genetic data to infer causality—provided evidence for the potential role of mechanisms related to inflammation, dyslipidemia, adiposity and acylcarnitine metabolisms (involved in mitochondrial fatty acids oxidation) in the development of overall depression and specific symptoms of fatigue, altered sleep and increased appetite. The abovementioned schematic scenarios and mechanisms are therefore not mutually exclusive, but rather belong to the same, complex pathophysiological background. Future mechanistic and experimental studies may elucidate the causal pathways involved in the link between metabolic signatures and specific depression clinical profiles.

The present findings are in line with previous evidence showing an association of immuno-metabolic markers with atypical/energy-related depressive symptoms. A previous study [16] in the NESDA cohort adopting a similar approach showed that the AES profile was specifically associated with an inflammatory index (integrating CRP and IL-6 levels), a metabolic syndrome index (integrating the five metabolic syndrome components) and their combination. Similarly, another NESDA analysis [17] showed that the AES symptom profile was associated with tryptophan catabolites of the indoleamine pathways “neurotoxic” branch, such as kynurenine and quinolinic acid. An analysis based on the large Netherlands Epidemiology of Obesity (NEO) study [13] examined the association of four adiposity indexes (BMI, waist circumference, percentage of body fat and visceral fat) with individual items of the IDS questionnaire. The four consistently strongest associations across all adiposity indexes were those with atypical energy-related symptoms of increased appetite, leaden paralysis, low energy level, and increased weight. In a joint NESDA/NEO meta-analysis [36], these AES symptoms were shown to be associated with a polygenic risk score (PRS) capturing the genetic risk for increased body fat accompanied by an unfavorable metabolic profile. In contrast, the AES profile was not associated with a PRS indexing the risk of increased body fat without metabolic alterations, suggesting that the established link between adiposity and AES profile emerges only in the presence of metabolic dysregulations, which may represent the connecting substrate between the two conditions.

This clustering between specific biological and clinical features has been postulated to identify a theoretical dimension labelled “immuno-metabolic depression (IMD)” [19], which does not represent and new established clinical entity, but rather a conceptual framework to explore depression heterogeneity and its commonality with other disorders. Biological (e.g. inflammatory, metabolic and neuroendocrine alterations) and clinical (e.g. atypical/energy-related symptoms) features clustering around the IMD axis are indeed partially shared with other transdiagnostic constructs such as sickness behavior or anhedonia [42–44], or with other psychiatric (e.g bipolar disorder, seasonal affective disorder) or somatic (e.g obesity) conditions. Aligning with the NIMH Research Domain Criteria (RDoC) framework [45], IMD may be conceptualized as a dimension in the heterogeneity space of depression mapping the degree of expression of behavioral and biological process overlapping with those in cardiometabolic phenotypes. Once better characterized this dimension may be empirically translated in tools identifying, for instance, depressed subjects at higher cardiometabolic risk. In a previous study [46] on >1000 subjects with major depression, a data-driven analyses derived a dimension underlying the co-covariance structure between multiple metabolites and depressive symptoms, characterized by relatively higher loading for symptoms like sleeping alterations, increased appetite and weight and low energy level. This dimension, transported in an independent population-based cohort, was significantly associated with higher markers or cardiometabolic risk such as triglycerides, insulin resistance and adiposity indexes. Consistently, large-scale epidemiological studies [16, 47–49] showed longitudinal associations between depression characterized by atypical-like symptoms and increase over time of cardiovascular risk factors and disease incidence. Furthermore, bio-clinical features clustering around IMD may be leveraged to guide the selection of depressed patients to be matched with treatments targeting related biological pathways [50]. For instance, a new generation of clinical trials [51–53] is testing anti-inflammatory (add-on) treatments in depressed subjects selected for features such as the presence of inflammation, somatic or atypical-energy related symptoms, or comorbidity with obesity. Results from these studies will provide key insights on the validity of this stratified approach and guidance for further development of this research framework.

A major limitation of the present study is the cross-sectional design of the analyses, which estimated the associations between metabolite levels and depressive symptoms measured at the same assessment (either baseline or 6-year follow-up), not allowing to draw conclusions on causality. The AES symptoms, selected based on theory and previous research results [19], were measured with items extracted from an existing questionnaire available in our cohort. The satisfactory mean inter-item correlation estimated, together with evidence [19] of consistent associations of the AES symptoms with immuno-metabolic benchmark markers, suggests a relative internal consistency of the score utilized. Nevertheless, future research examining the clinical dimensions of IMD should consider the adoption or development of dedicated instruments undergoing full psychometric study and validation. Furthermore, the sample combined different participants, including subjects with current and remitted depression and anxiety disorders and healthy controls. Nevertheless, the pattern of metabolite-symptom associations was substantially consistent in analyses focusing on a clinically selected and homogenous subsample of currently depressed cases compared to healthy controls. This consistency may support the hypothesis of a transdiagnostic process present in different degrees across different developmental psychopathological stages and various disorders [45]. Strengths of the current study are the large sample size and the detailed clinical assessment of depression and related characteristics. A unique stenght is represented by the internal replication of the main findings with data collected from the same subject (*N* > 2000) at a different time point, supporting the consistency and reliability of the associations detected. While replication data were collected in the same cohort, previous studies based on partially-related premises and methods showed replication of specific results—such as metabolite-symptom covariance decomposition [46] or the association between inflammatory markers and AES [18]—in independent samples. Nevertheless, the present findings warrant further external replication in independent samples.

To conclude, our study identified a specific metabolomic signature linked to a depression clinical profile characterized by atypical, energy-related depressive symptoms. Further independent replication and longitudinal analyses examining the directionality of this association are needed. The specific clustering of these metabolomic signature and clinical profile may identify a more homogenous subgroup of depressed patients at higher cardiometabolic risk. This may represent a valuable target for interventions aiming at reducing depression’s detrimental impact on health.

## Supplementary information

Supplemental materials

Supplemental tables

## Data Availability

R code used for analyses is available upon request to the authors.
