# Supplementary material for: Metabolomics signatures of depression: the role of symptom profiles
Source: Transl Psychiatry. 2023 Jun 10;13:198. doi: 10.1038/s41398-023-02484-5 (PMC10257720; doi:10.1038/s41398-023-02484-5)
Supplement: Supplementary file 1 — Supplemental materials [file 41398_2023_2484_MOESM1_ESM.docx]

**SUPPLEMENTARY INFORMATION**

**Metabolomics signatures of depression: the role of symptom profiles**

Table of Contents

[Supplementary methods 2](#_Toc126263635)

[Construction of ‘other-IDS symptoms severity’ 2](#_Toc126263636)

[Construction of ‘potential non-AES profiles’ and comparisons of statistical significance of their association with metabolites 2](#_Toc126263637)

[Supplementary Figures 3](#_Toc126263638)

[Supplementary Figure 1. Correlation between 31 metabolite-symptom association estimates at baseline and 6-year follow-up 3](#_Toc126263639)

[Supplementary Figure 2. Correlations between levels of the 31 metabolites associated with atypical, energy-related symptom profile 4](#_Toc126263640)

# ***Supplementary methods***

## **Construction of ‘other-IDS symptoms severity’**

In order to examine and compare specificity of findings for the AES profile, analyses were also conducted with an overall severity index of other depressive symptoms. For this purpose, a sum score of 25 individual IDS items other than the five items from the AES profile (other-IDS symptom severity) was constructed, which ranges from 0 (not severe) to 75 (severe). Prior to the summation, a missing individual item was imputed by the mean of the IDS items other than atypical, energy-related, only when the total number of missing IDS items per participant was < 6. When the total number of missing items per participant was ≥ 6, no sum score was created.

## **Construction of ‘potential non-AES profiles’ and comparisons of statistical significance of their association with metabolites**

There were 53,130 possible combinations of five IDS items other than those falling under AES, so 53 130 sum scores were created. Each sum score reflects a potential symptom profile that do not include any atypical, energy-related symptom (“potential non-AES profile”). For each of the potential non-AES profile separately, 51 linear regressions were conducted with the metabolomic marker as independent and the potential non-AES profile as dependent variable, while adjusting for all covariates of model 1. For the sake of checking the specificity of the associations between the metabolomic markers and the AES profile, we estimated FDRs for each metabolite associated with the AES profile. This reflects the chance that a metabolomic marker is associated with any of the 53 130 potential non-AES profiles at a lower *p* value than the one of the atypical, energy-related symptom profile. FDRs for metabolite i were calculated with the following formula:

FDR_(i)_= (number of *p* values_(potential non-AES profiles)_ ≤ *p* value_(i)_ /53,130) /

number of *p* values_(AES profiles)_ ≤ *p* value_(i)_ .

# ***Supplementary Figures***

## **Supplementary Figure 1. Correlation between 31 metabolite-symptom association estimates at baseline and 6-year follow-up**


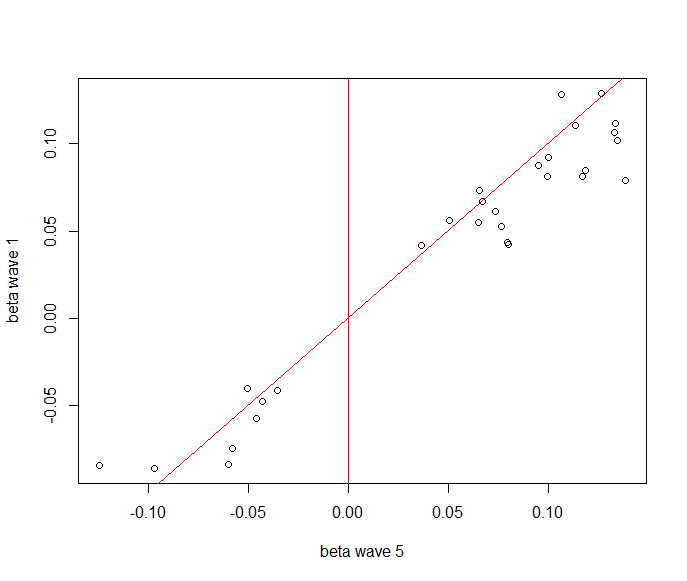


Estimates from linear regression models adjusted for age, sex, education level, smoking status, fasting status, number of somatic diseases, assessment batch, and lipid lowering drugs.

Wave1: baseline; wave 5: 6-year follow-up

## **Supplementary Figure 2. Correlations between levels of the 31 metabolites associated with atypical, energy-related symptom profile**


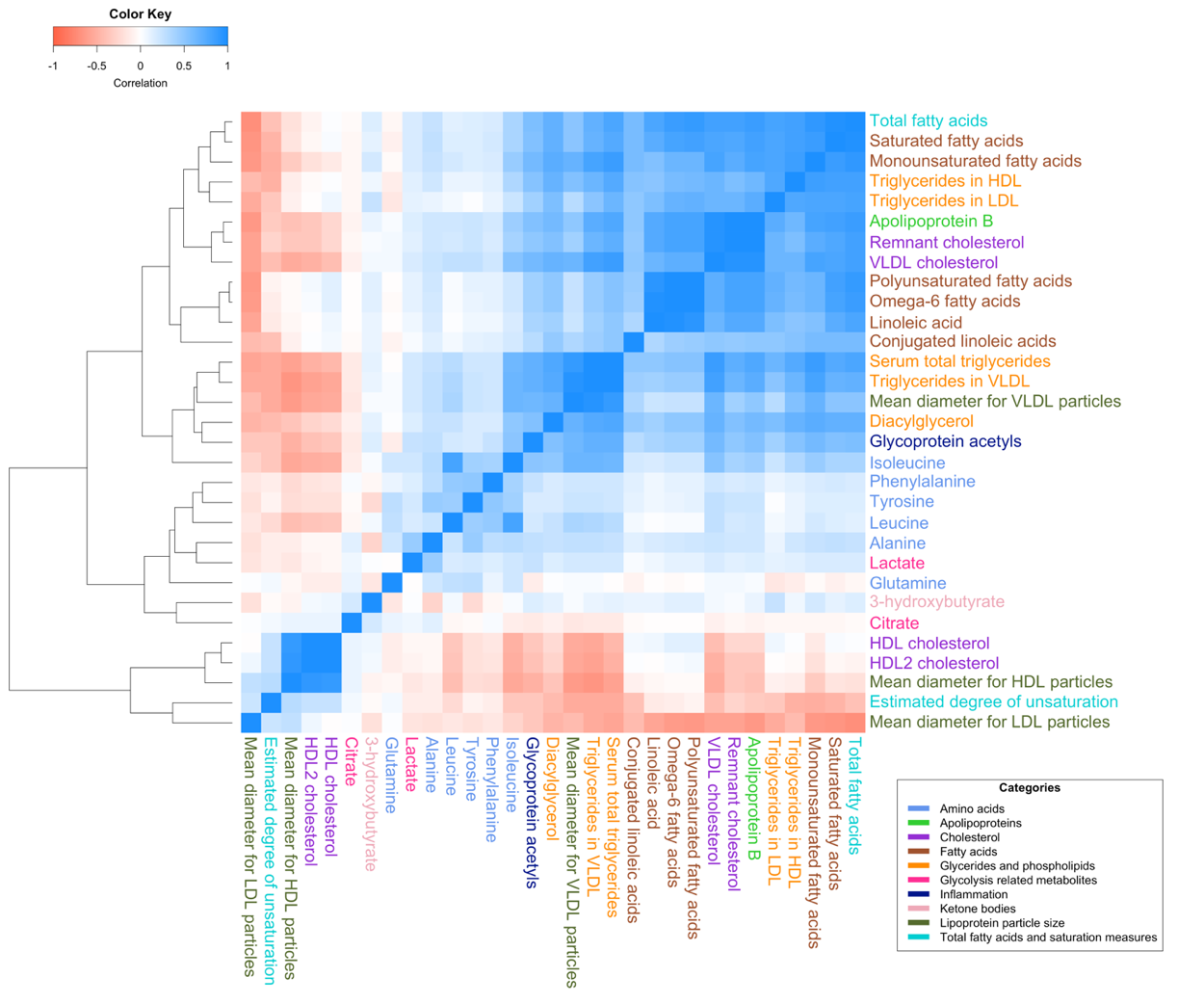


The colors of the markers’ names reflect the categories defined by Nightingale Health Ltd.
